# Chemical and flavor profile changes of cocoa beans (*Theobroma cacao* L.) during primary fermentation

**DOI:** 10.1002/fsn3.1701

**Published:** 2020-06-15

**Authors:** Yiming Fang, Rui Li, Zhong Chu, Kexue Zhu, Fenglin Gu, Yanjun Zhang

**Affiliations:** ^1^ Spice and Beverage Research Institute Chinese Academy of Tropical Agricultural Sciences Wanning China; ^2^ National Center of Important Tropical Crops Engineering and Technology Research Wanning China; ^3^ Hainan Provincial Engineering Research Center of Tropical Spice and Beverage Crops Wanning China

**Keywords:** change of quality, chemical and flavor profile, cocoa beans (*Theobroma cacao* L.), fermenting process, quality evaluation

## Abstract

This survey reports for the first time the changed of quality of fermented cocoa (*Theobroma cacao* L.) beans. The quality evaluation and simultaneous detection of amino acids, flavor, procyanidin, color, fat, protein, antioxidant activity, and enthalpy were obtained for different fermentation stages of cocoa beans. The results showed that total essential amino acids contents ranged from 2.64 g/100 g to 3.68 g/100 g. A total of 88 compounds identified at the end of the fermentation belonged to alcohols, acids, esters, ketones, pyrazines, aldehydes, and terpenoids. One of the chemical groups that were present in highest abundance in the consummation treatments was acids, representing 56.04% of the total extracted area, followed by alcohols (22.95%) and ketones (9.40%). The colors of the beans in different fermentation stages were different, from deep purple to deep red‐brown. Fermented cocoa beans were shown to be 53.45% and 13.51% bean butter and protein content, respectively. The value of denaturation enthalpy (Δ*H*) ranged from 30.4 (J/g) to 43.38 (J/g). The 3‐day fermented sample had the highest Δ*H* (43.38 J/g). When the fermentation process was complete, the procyanidin concentration of the beans decreased, with the final yield of procyanidin at 6.2%. During fermentation, the antioxidant capacity of beans gradually reduced. The fermenting of cocoa beans had a significant effect on the quality formation. The findings of this study constitute a basis for further investigations on the quality formation of cocoa during fermentation.

## Graphical abstract

This survey reports for the first time the changed of quality of fermented cocoa beans. The quality evaluation and simultaneous detection of amino acids, flavor, procyanidin, color, fat, protein, antioxidant activity, and enthalpy were obtained for different fermentation stages of cocoa beans. The fermenting of cocoa beans had a significant effect on the quality formation.

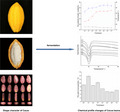

## 1 INTRODUCTION

Cacao beans (*Theobroma cacao* L.) are the primary raw material of chocolate manufacture. Raw cacao has an astringent, unpleasant taste, and flavor. Studies have shown that unfermented cacao beans do not contain the aroma precursors that are present in the fermented cocoa beans (Cevallos‐Cevallos, Gysel, Maridueña‐Zavala, & Molina‐Miranda, 2018). To obtain the characteristic cocoa flavor and color, raw cacao must be fermented (Gu et al., 2013). Fermentation of the cacao beans is necessary for removing the pulp that envelops the beans and becoming precursors of chocolate flavor (Batista, Ramos, Ribeiro, Pinheiro, & Schwan, 2015). If the fresh beans were dried without any fermentation, the beans would be salty and gray in color, rather than brown or purple‐brown, which is the color of fermented dried cocoa beans. Fermentation is a major step in developing specific volatile fractions, such as alcohols, esters, and acids. Fermentation is also necessary for the development of chocolate flavor and aroma precursors, such as amino acids and fat.

Normally, the cacao beans are placed in wooden fermenting boxes after harvesting, then covered with banana leaves and left to ferment. The fermentation time varies depending on the variety of the cacao beans (Afoakwa, Paterson, Fowler, & Ryan, 2008). Organic acid, formed during the fermentation process, is permeates in the cacao seed tissue so that a rise of the temperature in the fermentation mass is taken place, leading to the degradation of the cacao tissue by decomposition of proteins to flavor precursors like amino acids (Chetschik et al., 2018). As the fermentation process continues, the heat increases (up to 122°F), which causes the pulp to liquefy and drain off (Kresnowati & Febriami, 2015). The microorganism fermentation produces acids and alcohols that will also penetrate the cacao bean and start the chemical reactions that will form the precursors of chocolate flavor (Cempaka, Aliwarga, Purwo, & Kresnowati, 2014). However, few reports have been reported on the effects of fermentation on cocoa flavor. Moreover, none of the reported investigations involve the effects of fermentation on the changes of cocoa amino acids, color, enthalpy, and fat and so on.

Cocoa beans contain polyphenolic compounds, which can be further classified as flavonoids (Othman, Ismail, Abdul Ghani, & Adenan, 2007). The classes of flavonoids that are most commonly found in cocoa beans are flavan‐3‐ols, catechins, epicatechins, and proanthocyanidins, with a higher content of procyanidins. The flavonoids are important because they offer potential cardiovascular health benefits, antioxidant protections, and help balance cholesterol in the body (Harrington, 2011). Procyanidins, or condensed tannins, are dimmers, oligomers, and polymers of catechins. These procyanidins are responsible for the bitterness in chocolate (Fayeulle et al., 2018). However, there are no clear reports on the effects of fermentation on the changes of cocoa procyanidin and antioxidant activity. The software program of differential scanning calorimetry (DSC) was used to analyze and plot the thermal data (Falcaorodrigues, Moldaomartins, & Beiraodacosta, 2007). The enthalpy can be used to reflect the earlier responses to the physiological changes in cocoa beans. Differences in the thermograms are observed by the application of fermenting cocoa beans.

Therefore, in this study, Hainan cocoa beans are used as raw materials, and this study aims to investigate the following: (a) quality variation of Hainan cocoa beans as a result of the fermentation process and (b) the content and composition of amino acids, flavor, and other factors found in fermented cocoa so that variations in fermented cocoa beans from different stages can be described.

## 2 MATERIALS AND METHODS

### 2.1 Sample preparation

Cacao beans were harvested from the plant garden of the Spice and Beverage Research Institute and China Academy of Tropical Agricultural Science (Wanning, China), were then fermented by wooden box, and were marked as 0/7 to 7/7, respectively. The wooden box was filled with cacao beans; exit holes or grooves, usually on the box floor, provided ventilation and permitted fluids and pulp to drain away. Exit holes were on the end of the plate, and the top of the plate was covered with sackcloth. Freshly harvested beans of 50 kg were used for fermentation. The samples were prepared as reported (Gutiérrez, 2017). The wooden box (0.8 m × 0.8 m × 0.8 m) was supplied by the Spice and Beverage Research Institute, and the cacao beans were harvested in the same season and maturity. The fermentation of the material started spontaneously, and the material was stirred every 24 hr with a wooden shovel. Once the fermentation was complete, the beans were dried in the sun. After seven‐day fermentation, the cocoa beans were collected and dried until 4% moisture content.

### 2.2 Measurement of amino acids

The ninhydrin was obtained from Aladdin Co. (Shanghai, China, purity >98%), mixed amino acids standard and 16 amino acid standards were purchased from Sigma‐Aldrich Co. (Shanghai, China, purity >98%); hydrochloric acid, phenol, sodium citrate, and sodium hydroxide were of analytical grade and were obtained from Sinopharm Co. Hydrolysis amino acids were measured as reported (Noor‐Soffalina, Jinap, Nazamid, & Nazimah, 2009). Total content of amino acids was determined by ninhydrin method. The defatted residue or cocoa powder was hydrolyzed with 6 M hydrochloric acid at 110°C for 24 hr under vacuum (Gu et al., 2013). The supernatant was filtered through a 0.22 μm nylon filter membrane and subjected to an automatic amino acid analyzer (Sykam Ltd, S‐433D). The content of each amino acid was determined as described by Chen et al. (Chen et al., 2017). The flow rate of the mobile phase was 0.45 ml/min, and the flow rate of the derivatizating reagent was 0.25 ml/min. The column temperature was kept at 57–74°C, and the postcolumn reaction equipment was set at 130°C. The injection volume was 50 μl. The amino acid constituents of cocoa beans were analyzed by different stages of fermentation for the seven days. Only proline acid was detected at 440 nm, and the other amino acids were detected at 570 nm.

The content of each amino acid of mixed amino acids in the standard stock solution was determined according to formula (1): *c_j_*: The concentration of *j* amino acid of mixed amino acids in the standard stock solution; (2): *c_i_*: The content of *i* amino acids in the sample test solution. *A_i_*: The peak area of *i* amino acid in the sample test solution. *A_s_*: The peak area of *s* amino acids in the standard working fluid. *c_s_*: The content of *s* amino acids in the standard working fluid. The content of each amino acid in the sample was calculated according to the formula; (3): *X_i_*: The content of *i* amino acids in the sample. *c_i_*: The content of *i* amino acids in the sample test solution.

(1) $$c_{j}=\frac{m_{j}}{M_{j}\times 250}\times 1000,$$

(2) $$c_{i}=\frac{C_{s}}{A_{s}}\times A_{i},$$

(3) $$X_{i}=\frac{C_{i}\times F\times V\times M}{m\times 10^{9}}\times 100$$

### 2.3 Headspace GC‐MS conditions

The GC‐MS was carried using the modified method by Olivares et al (Olivares et al., 2011). Unfermented cacao beans from raw beans were marked as 0/7, and fermented cocoa beans from different fermentation days were marked as 1/7 to 7/7, respectively. One gram of cocoa powder was placed in the sample cylinder to extract aroma compounds using the headspace solid‐phase microextraction (SPME) method. The extracted time, temperature, and type of head were 50 min, 60°C, and divinylbenzene/ carboxen/ polydimethylsiloxane (DVB/CAR/PDMS, 50 mm) fiber, respectively. Mass spectra data were obtained with Agilent GC‐MS. The ion source temperature was 250°C, the electric quadrupole temperature was 150ºC, the transfer line temperature was 240°C, the full‐scan mass range was 35–500, the capillary column was TR‐WAXMS (30 m × 0.25 mm, 0.25 μm), the oven temperature was 50°C for 2 min, with 2°C/min to 180°C, retaining for 1 min, and then 10°C/min to 230ºC, retaining for 2 min, with a constant flow rate of 1.0 ml/min. Identification method was compared with Nist05, Mainlib and Replib Mass Spectral Database. The relative content was calculated as a percentage.

### 2.4 Measurement of bean temperature, color, fat, and protein content

The beans were lyophilized using a vacuum freeze dryer (Scientz‐18ND, Biological Technology Co., Ningbo, China). Bean color was measured using an x‐rite spectrophotometer (Xrite Inc.). The cocoa beans from different days of fermentation were placed under a spectrophotometer, and the values of sample luminance L* (quantity of reflected light), chromatic coordinated a* (red‐green axis), and b* (yellow‐blue axis) were measured. This process was repeated three times for each cocoa bean in which a color can be defined conventionally using the method by Gu et al. (Gu et al., 2013). Cocoa bean shells were removed, the lyophilized dry cocoa beans were crushed, and 3.0 g portions were extracted repeatedly in the Soxhlet extractors with 250 ml of petroleum ether (b.p. 30–60°C) (Kratzer et al., 2009). Protein was measured using the Dumas nitrogen analyzer (Velp, Italy) (Kim et al., 2008). Fat and protein contents were expressed as a percentage of weight. The internal temperature of the beans in the box was measured at the same time each day.

### 2.5 Measurement of enthalpy

The thermal characteristics of the cocoa beans were analyzed using a differential scanning calorimeter (DSC‐Q2000). The equipment was calibrated with indium and zinc, and an empty, sealed but pierced aluminum pan was used for reference. Onset temperature (*T*_o_), peak or denaturation temperature (*T*_d_), peak temperature (*T*_p_), and denaturation enthalpy (Δ*H*) were determined using the software PLATIUM, Universal V4.5A. Detection of DSC was carried out using previously reported procedures (Colombo, Ribotta, & Leon, 2010), with slight modifications. Cocoa beans were fermented for seven days (Section 2.1), and the samples were marked as 0 to 7 days, respectively. Cocoa powder (6 mg) was placed in aluminum pans. The fermented cocoa beans obtained were dispersed (1:2, w/w) in distilled water. Pans were hermetically sealed, incubated at room temperature for 24 hr, and scanned at 10°C/min from 10 to 100°C.

### 2.6 Measurement of procyanidin

Procyanidin and vanillin were purchased from Shanghai Chemical Reagent Co. (Shanghai, China, purity >98%). The content of procyanidin in the extract solution was determined using the standard vanillin–HCl method (Ma, Zhou, Qiang, & Zhang, 2014). The cocoa powder was lyophilized and degreased. A portion of the powder was dissolved in extraction solvent and heated in a 76°C water bath for 88 min. The ratio of materials to solvent was 1:20. The supernatant was filtered through a 0.45 μm nylon filter membrane. Vanillin/methanol solution (40 mg/ml) of 3 ml and concentrated HCl of 1.5 ml were added to 1.0 ml of the extract solution in a brown volumetric flask, methanol was replenished to 10 ml, and then the flask was capped immediately. The solution was mixed well and incubated at 20°C (±1°C) in an electrically heated water bath for 20 min. The procyanidin content was calculated according to the standard curve constructed for procyanidin, the concentration of the reference substance (mg/ml) within a concentration range of 0.004–0.1 mg/ml. The changes in the levels of procyanidins during fermentation were determined at 500 nm using a UV‐Vis Analytik Jena Specord 250 PLUS spectrophotometer (Germany).

### 2.7 Measurement of antioxidant activity

The diammonium salt of 2, 2′‐azinobis (3‐ethylbenzothiazoline‐6‐sulfonate) (ABTS) was purchased from Aladdin, and 2, 2′‐diphenyl‐1‐picrylhydrazyl (DPPH) was purchased from Sigma (purity > 98%). The concentration of DPPH and ABTS^＋^ remaining after reaction with the antioxidants was determined according to previously published methods (Tang & Liu, 2007). 1 g of cocoa powder was vortex extracted with ethanol (20 ml, 90%, 2,000 rmp) for 5 min. The extracts were ultrasonic extracted, under water bath at 50°C for 1 hr. The extracts were centrifuged at 4,000 r/min for 10 min, and supernatant was collected. Residue was extracted twice with ethanol. Extracts were concentrated using rotated evaporator and volume to 25 ml with methanol (90%, v/v). DPPH was dissolved in ethanol in sufficient concentration to produce a solution with an absorbance at 517 nm (*Abs*_ref_). The procedure to prepare the ABTS^＋^ stock solution was modified slightly. Sufficient amounts of the diammonium salts of ABTS and K_2_S_2_O_8_ were dissolved in 10 ml water to achieve concentrations of 7.00 and 2.45 mM, respectively. This solution was kept in the dark for at least 16 hr to form ABTS^＋^, then diluted to 100 ml with ethanol so that the solution had an absorbance or *Abs*_ref_ of ~0.70 at 734 nm. Various concentrations of solution were added to DPPH or ABTS^＋^ solution at ambient temperature to reach a stable absorbance (*Abs*_detect_). Then, the percentage of DPPH (or ABTS^＋^) scavenged was calculated according to the formula: $\text{Scavenging}\;\text{DPPH}\;\left(\text{or}\;{\text{ABTS}}^{+\cdot }\right)\left(\%\right)=\left(1-\frac{Abs_{\text{detect}}}{Abs_{\text{ref}}}\times 100\right)$ .

### 2.8 Statistical analysis

All tests were performed in triplicate and expressed as the mean value ± standard deviation. The experimental data were analyzed using analysis of variance (ANOVA) and computed using the commercial statistical software, SPSS 16.0. The graphics were draw using the Origin Pro 9.1 software (Origin Lab). The standard deviation and significant differences among the mean values were received by Duncan's test at *p* < 0.05.

## 3 RESULTS AND DISCUSSION

### 3.1 Effect of the fermentation on the total and essential amino acids content

Aroma precursors in cocoa beans, which include free amino acids and protein, develop into a cocoa‐specific aroma through fermentation (Gu et al., 2013). Effects of fermentation on the total hydrolyzed and essential amino acids of cocoa beans are shown in Table 1.

**Table 1:** Amino acids changes of cocoa beans during primary fermentation

Amino acids (%)	0/7	1/7	2/7	3/7	4/7	5/7	6/7	7/7
ASP	1.043 ± 0.011^b^	1.071 ± 0.003^a^	0.995 ± 0.006^c^	0.953 ± 0.012^d^	0.850 ± 0.008^e^	0.806 ± 0.009^f^	0.786 ± 0.006^g^	0.762 ± 0.006^h^
THR*	0.416 ± 0.005^a^	0.418 ± 0.001^a^	0.393 ± 0.002^b^	0.376 ± 0.006^c^	0.339 ± 0.003^d^	0.311 ± 0.012^e^	0.292 ± 0.003^f^	0.289 ± 0.003^f^
SER	0.525 ± 0.007^a^	0.513 ± 0.003^b^	0.477 ± 0.001^c^	0.453 ± 0.009^d^	0.409 ± 0.005^e^	0.339 ± 0.003^f^	0.312 ± 0.003^g^	0.321 ± 0.008^g^
GLU	1.803 ± 0.021^b^	1.878 ± 0.014^a^	1.683 ± 0.008^c^	1.610 ± 0.015^d^	1.479 ± 0.016^e^	1.434 ± 0.013^f^	1.394 ± 0.011^g^	1.387 ± 0.010^g^
GLY	0.493 ± 0.006^a^	0.492 ± 0.0003^a^	0.472 ± 0.014^b^	0.444 ± 0.005^c^	0.400 ± 0.003^d^	0.379 ± 0.003^e^	0.376 ± 0.003^e^	0.355 ± 0.003^f^
ALA	0.529 ± 0.007^a^	0.508 ± 0.001^b^	0.486 ± 0.008^c^	0.459 ± 0.004^d^	0.415 ± 0.003^e^	0.395 ± 0.004^f^	0.381 ± 0.004^g^	0.361 ± 0.003^h^
VAL*	0.613 ± 0.008^a^	0.613 ± 0.005^a^	0.587 ± 0.003^b^	0.563 ± 0.006^c^	0.510 ± 0.005^d^	0.496 ± 0.005^e^	0.496 ± 0.003^e^	0.472 ± 0.004^f^
MET*	0.098 ± 0.002^b^	0.160 ± 0.002^a^	0.069 ± 0.001^c^	0.063 ± 0.001^d^	0.061 ± 0.002^d^	0.055 ± 0.001^e^	0.061 ± 0.002^d^	0.057 ± 0.001^e^
ILE*	0.409 ± 0.005^a^	0.412 ± 0.004^a^	0.397 ± 0.002^b^	0.379 ± 0.005^c^	0.349 ± 0.004^d^	0.336 ± 0.003^e^	0.335 ± 0.001^e^	0.322 ± 0.003^f^
LEU*	0.725 ± 0.009^a^	0.729 ± 0.004^a^	0.699 ± 0.004^b^	0.661 ± 0.010^c^	0.598 ± 0.005^d^	0.568 ± 0.006^e^	0.560 ± 0.003^e^	0.534 ± 0.004^f^
TYR	0.460 ± 0.008^a^	0.428 ± 0.009^b^	0.431 ± 0.003^b^	0.401 ± 0.006^c^	0.369 ± 0.005^c^	0.340 ± 0.009^d^	0.343 ± 0.001^d^	0.333 ± 0.003^d^
PHE*	0.634 ± 0.009^a^	0.625 ± 0.005^a^	0.593 ± 0.005^b^	0.559 ± 0.012^c^	0.496 ± 0.006^d^	0.468 ± 0.005^e^	0.463 ± 0.002^e^	0.433 ± 0.004^f^
HIS	0.205 ± 0.002^a^	0.204 ± 0.003^a^	0.201 ± 0.001^a^	0.188 ± 0.003^b^	0.181 ± 0.002^c^	0.176 ± 0.002^d^	0.173 ± 0.003^d^	0.164 ± 0.001^e^
LYS*	0.707 ± 0.008^b^	0.718 ± 0.004^a^	0.670 ± 0.003^c^	0.631 ± 0.008^d^	0.568 ± 0.005^e^	0.549 ± 0.005^f^	0.543 ± 0.004^f^	0.531 ± 0.004^g^
ARG	0.807 ± 0.008^a^	0.772 ± 0.003^b^	0.716 ± 0.029^c^	0.649 ± 0.005^d^	0.579 ± 0.009^e^	0.552 ± 0.007^f^	0.547 ± 0.004^f^	0.541 ± 0.004^f^
PRO	0.482 ± 0.004^ab^	0.484 ± 0.011^a^	0.465 ± 0.010^b^	0.448 ± 0.005^c^	0.412 ± 0.010^d^	0.389 ± 0.007^e^	0.397 ± 0.012^de^	0.372 ± 0.014^f^
EAA	3.602	3.675	3.408	3.232	2.921	2.783	2.75	2.638

Note

ASP is aspartic acid; THR is threonine; SER is serine; GLU is glutamic acid; GLY is glycine; ALA is alanine; VAL is valine; MET is methionine; ILE is isoleucine; LEU is leucine; TYR is tyrosine; PHE is phenylalanine; HIS is histidine; LYS is lysine; ARG is arginine; PRO is proline. “EAA” is abbreviation for essential amino acid.

It was found that cocoa beans by 0/7 and 1/7 had the highest concentrations of essential amino acids. The total essential amino acid content of cocoa beans by the 7/7 was the lowest (2.64%). Cocoa beans processed by the 0/7 were found to have approximately 0.964% higher total essential amino acid contents than those of the fermented. The total hydrolyzed amino acids contents ranged from 7.23 g/100 g to 10.03 g/100 g. The 7/7 fermented sample had the lowest total hydrolyzed amino acid content. These results were lower than the value of 14.51 g/100 g reported by Gu, et al (Gu et al., 2013). The differences in values may be due to differences in measurement, as the content of un‐defatted beans was tested in the current study, while the defatted beans were tested in the above literature. Also, presumably the fermentation environment used on cocoa bean influences the composition and content of the amino acids. The trend of change is different from the reports (Rohsius, Matissek, & Lieberei, 2006) (Tchouatcheu, Noah, Lieberei, & Niemenak, 2019). The differences in changes may be due to differences in process; Rohsius et al. (2006) describes a comparative study on fermented (but not dried) cocoa beans. Tchouatcheu et al. (2019) describes that the sample was dried under vacuum at room temperature. This research describes a comparative study on fermented (dried) cocoa beans. Amino acids are converted to the typical cocoa aroma via Strecker degradation during the drying process (heat air drying at 60℃ in this article). The applied heating reaction may influence the final amount of total amino acids of cocoa. The large numbers of free amino acids could be degraded by Strecker degradation (Zhou et al., 2020). Furthermore, free amino acids were measured in references, and the hydrolysis amino acids (total amino acids) were measured in this article.

### 3.2 Identification of the key aroma compounds in cocoa bean based on sensory correlations

Fermentation is an important processing step for developing cocoa. To study the effect of fermentation day on the processes involved in the development of the aroma compounds, it is necessary to identify the volatile compounds according to their possible origins (Hinneh et al., 2018). Aroma was reported to play a significant role in the quality of cocoa beans (Frauendorfer & Schieberle, 2008). The volatile compounds were extracted by SPME and were analyzed by GC‐MS (Tables 2 and 3). A total of 88 compounds identified at the end of the process belonged to alcohols, acids, esters, ketones, pyrazines, aldehydes, and terpenoids. Volatile compounds are always present in the fermentation process.

**Table 2:** Relative contents of major flavor profile compounds of cocoa beans during the fermentation

Flavor	0/7	1/7	2/7	3/7	4/7	5/7	6/7	7/7
Alcohol								
2‐pentanol	9.204 ± 0.811^b^	33.077 ± 2.792^a^	1.539 ± 0.417^c^	8.707 ± 2.097^b^	0.407 ± 0.282^c^	0.972 ± 0.429^c^	1.136 ± 0.559^c^	1.049 ± 0.290^c^
2‐methyl−1‐propanol	4.336 ± 0.656^a^	**–**	1.153 ± 0.291^b^	0.154 ± 0.069^c^	**–**	0.025 ± 0.006^c^	0.404 ± 0.030^c^	0.110 ± 0.031^c^
3‐methyl−1‐butanol	26.066 ± 0.845^a^	17.445 ± 2.120^b^	18.592 ± 0.540^b^	2.524 ± 0.182^c^	0.851 ± 0.241^d^	0.394 ± 0.016^d^	0.338 ± 0.066^d^	1.704 ± 0.710^cd^
2,3‐butanediol	15.848 ± 2.409^b^	10.238 ± 3.374^c^	16.274 ± 1.454^b^	17.581 ± 0.357^ab^	11.029 ± 0.559^c^	4.347 ± 0.494^d^	15.559 ± 0.584^b^	19.333 ± 0.745^a^
Benzyl alcohol	0.085 ± 0.018^ab^	0.055 ± 0.008^c^	0.074 ± 0.004^abc^	0.064 ± 0.008^bc^	0.075 ± 0.025^abc^	0.070 ± 0.005^abc^	0.085 ± 0.007^ab^	0.088 ± 0.006^a^
Phenylethyl alcohol	1.961 ± 0.931^b^	1.357 ± 0.521^bc^	4.348 ± 0.274^a^	1.868 ± 0.615^b^	1.590 ± 0.303^b^	0.595 ± 0.037^cd^	0.484 ± 0.091^d^	0.661 ± 0.069^cd^
2‐heptanol	0.401 ± 0.224^c^	2.652 ± 1.066^a^	1.834 ± 0.066^ab^	2.261 ± 1.064^a^	1.096 ± 0.300^bc^	0.920 ± 0.272^bc^	0.378 ± 0.114^c^	**–**
3‐methyl−2‐butanol	**–**	**–**	25.468 ± 0.210^a^	8.707 ± 2.097^b^	4.555 ± 0.765^c^	1.187 ± 0.096^d^	**–**	**–**
Acid								
Acetic acid	12.966 ± 0.306^d^	4.090 ± 0.480^e^	8.084 ± 0.720^e^	31.827 ± 5.629^c^	46.569 ± 3.814^a^	50.530 ± 1.631^a^	41.390 ± 2.213^b^	33.897 ± 1.634^c^
Pentanoic acid	0.087 ± 0.055^b^	**–**	**–**	0.113 ± 0.030^ab^	0.185 ± 0.092^ab^	0.169 ± 0.018^ab^	0.193 ± 0.011^a^	**–**
2‐methyl‐propanoic acid	0.685 ± 0.063^e^	0.220 ± 0.035^e^	0.620 ± 0.041^e^	3.203 ± 0.623^d^	3.868 ± 0.113^c^	0.137 ± 0.003^e^	6.440 ± 0.517^b^	8.543 ± 0.588^a^
Hexanoic acid	0.277 ± 0.039^bc^	0.258 ± 0.059^c^	0.188 ± 0.025^d^	0.342 ± 0.028^b^	0.419 ± 0.038^a^	0.293 ± 0.020^bc^	0.478 ± 0.022^a^	**–**
Butanoic acid	**–**	0.087 ± 0.055^d^	0.194 ± 0.007^c^	0.165 ± 0.014^c^	0.199 ± 0.049^c^	0.220 ± 0.007^c^	0.310 ± 0.005^b^	0.455 ± 0.051^a^
3‐methyl‐butanoic acid	1.787 ± 0.250^e^	0.762 ± 0.108^f^	3.076 ± 0.038^d^	1.522 ± 0.265^e^	2.888 ± 0.148^d^	6.427 ± 0.252^c^	9.056 ± 0.842^b^	12.906 ± 0.647^a^
Octanoic acid	0.064 ± 0.011^b^	**–**	**–**	**–**	0.121 ± 0.019^a^	0.070 ± 0.008^b^	0.135 ± 0.014^a^	0.059 ± 0.011^b^
Benzoic acid	0.262 ± 0.029^a^	**–**	**–**	0.476 ± 0.237^a^	0.311 ± 0.116^a^	0.330 ± 0.251^a^	0.290 ± 0.017^a^	0.183 ± 0.053^a^
Ester								
1‐butanol, 3‐methyl‐, acetate	1.945 ± 0.647^a^	**–**	**–**	**–**	**–**	0.717 ± 0.072^b^	0.012 ± 0.005^c^	0.276 ± 0.152^bc^
Benzeneacetic acid, ethyl ester	**–**	**–**	0.041 ± 0.006^b^	0.112 ± 0.024^a^	0.114 ± 0.044^a^	0.072 ± 0.005^ab^	0.078 ± 0.011^ab^	**–**
Butyrolactone	4.450 ± 0.161^a^	1.605 ± 0.769^b^	0.507 ± 0.021^c^	0.277 ± 0.026^c^	0.354 ± 0.116^c^	0.275 ± 0.023^c^	0.263 ± 0.090^c^	0.348 ± 0.038^c^
Formic acid, 1‐methylpropyl ester	0.271 ± 0.103^a^	**–**	**–**	**–**	**–**	**–**	0.003 ± 0.001^b^	0.328 ± 0.001^a^
Butanoic acid, 3‐hydroxy‐, ethyl ester	**–**	**–**	0.028 ± 0.011^b^	0.120 ± 0.018^a^	0.122 ± 0.025^a^	0.031 ± 0.004^b^	**–**	**–**
Butanoic acid, 2‐methyl−3‐oxo‐, methyl ester	**–**	**–**	**–**	**–**	0.072 ± 0.003^b^	0.182 ± 0.021^a^	0.088 ± 0.024^b^	**–**
2‐pentanol, acetate	**–**	**–**	1.206 ± 0.607^a^	0.938 ± 0.372^ab^	0.497 ± 0.147^bc^	0.280 ± 0.056^c^	0.241 ± 0.097^c^	**–**
2‐furancarboxylic acid, 2‐phenylethyl ester	**–**	**–**	0.179 ± 0.036^c^	0.495 ± 0.140^b^	0.744 ± 0.185^a^	0.370 ± 0.014^bc^	0.229 ± 0.002^c^	**–**
Ketones								
3‐hydroxy−2‐butanone	5.005 ± 0.380^c^	2.859 ± 1.144^d^	3.379 ± 0.156^d^	3.196 ± 0.387^d^	4.749 ± 0.999^c^	10.323 ± 0.914^a^	10.017 ± 0.596^a^	8.377 ± 0.903^b^
2‐heptanone	0.441 ± 0.165^d^	2.336 ± 0.893^a^	1.364 ± 0.089^b^	1.237 ± 0.438^bc^	0.461 ± 0.083^d^	0.529 ± 0.054^d^	0.487 ± 0.085^d^	0.705 ± 0.038^cd^
5‐methyl−2‐hexanone	**–**	**–**	**–**	0.523 ± 0.179^a^	0.461 ± 0.083^a^	0.271 ± 0.029^b^	0.003 ± 0.001^c^	**–**
4‐s‐butoxy−2‐butanone	**–**	**–**	**–**	0.056 ± 0.011^a^	**–**	0.064 ± 0.003^a^	0.073 ± 0.005^a^	**–**
Acetophenone	**–**	0.283 ± 0.058^b^	0.380 ± 0.031^ab^	0.386 ± 0.046^ab^	0.472 ± 0.186^a^	0.288 ± 0.008^b^	0.335 ± 0.042^ab^	0.318 ± 0.066^ab^
Pyrazines								
Trimethyl‐ pyrazine	**–**	**–**	**–**	0.240 ± 0.053^c^	0.662 ± 0.069^a^	0.364 ± 0.035^b^	0.251 ± 0.014^c^	0.082 ± 0.017^d^
2,3‐dimethyl‐ pyrazine							0.129 ± 0.003	
Tetramethyl‐ pyrazine	0.234 ± 0.064^c^	**–**	**–**	0.896 ± 0.199^b^	1.961 ± 0.411^a^	0.877 ± 0.044^b^	0.781 ± 0.038^b^	0.214 ± 0.093^c^
Other compounds								
Phenol	0.024 ± 0.005^b^	0.049 ± 0.050^b^	**–**	0.039 ± 0.006^b^	0.034 ± 0.004^b^	**–**	**–**	0.629 ± 0.065^a^
Benzaldehyde	0.240 ± 0.100^d^	0.267 ± 0.059^d^	0.915 ± 0.065^c^	2.386 ± 0.656^a^	1.945 ± 0.110^ab^	1.839 ± 0.138^b^	2.350 ± 0.056^a^	1.761 ± 0.184^b^
(2‐ethoxy−1‐methoxyethoxy)‐ ethene	**–**	**–**	0.208 ± 0.018^a^	0.182 ± 0.037^a^	**–**	**–**	0.106 ± 0.004^b^	**–**
Trimethyl‐oxazole	**–**	**–**	**–**	**–**	0.754 ± 0.042^a^	0.264 ± 0.022^b^	0.261 ± 0.017^b^	**–**
Acetamide	**–**	0.044 ± 0.009^d^	0.029 ± 0.003^d^	0.160 ± 0.025^ab^	0.189 ± 0.028^a^	0.160 ± 0.025^ab^	0.104 ± 0.005^c^	0.151 ± 0.016^b^
1,1‐dimethyl‐ hydrazine	0.277 ± 0.070^b^	**–**	**–**	**–**	**–**	**–**	0.688 ± 0.045^a^	0.222 ± 0.004^b^
5‐amino−3‐phenylpyrazole	**–**	**–**	**–**	**–**	0.023 ± 0.014^a^	0.013 ± 0.003^a^	0.020 ± 0.001^a^	**–**

Note

“**–**” Not detected.

**Table 3:** The comparison analysis of compounds before and after fermentation

Unfermented				
1. 1‐hexanol	2. 2‐heptanol	3. 3‐hexen−1‐ol	4. 2‐ethyl−1‐hexanol	5. 1‐butanol
6. 1‐butanol, 3‐methyl‐, acetate	7. 3‐methyl−3‐buten−1‐ol	8. 3‐methyl−2‐hexanol	9. 2‐methyl−1‐propanol	10. pentanoic acid
11. hexanoic acid	12. 2‐methyl‐butanoic acid	13. 2‐hydroxy‐propanoic acid, ethyl ester	14. 3‐methyl‐butanoic acid, ethyl ester	15. sulfurous acid, dipropyl ester
16. 2,3‐dioxo‐butyric acid, 2‐methyloxime, ethyl ester	17. decanoic acid, ethyl ester	18. trimethylene glycol monomethyl ether	19. tetrahydro−2‐methyl‐furan	20. *N*‐hydroxymethylacetamide
21.1,2‐dimethyl‐hydrazine	22. 2‐butanol	23. 1‐amino−2‐butanol	24.2‐[2‐(2‐ethoxyethoxy)ethoxy]‐ ethanol	25.4‐methoxy‐butanoic acid
26. 2‐methyl‐butanoic acid, ethyl ester	27. sec‐butyl nitrite	28. 1H‐pyrrole−2‐carboxaldehyde	29. 2‐furancarboxylic acid, 2‐phenylethyl ester	30. ethyldimethyl‐silane
31. 4‐methoxy−1‐butanamine	32. dimethyl sulfone			
Fermented				
33. 2‐hexanol	34. octaethylene glycol	35.3‐(1‐methylbutoxy)−2‐butanol	36. 2,3‐hexanediol	37. propanoic acid
38. butanoic acid	39. 3‐hydroxy‐butanoic acid	40. 2,4‐dimethyl‐hexanoic acid, methyl ester	41. 3‐methyl−3‐butenoic acid	42. 3‐methyl−2‐butenoic acid
43. 1,3‐butanediol, diacetate	44. 4‐methyl−2‐pentyl acetate	45. 3‐hydroxy−2‐butanone, acetate	46. 2‐hexanol, acetate	47.1‐(1,3‐dioxolan−2‐yl)−2‐propanone
48. 2‐nonanone	49. acetophenone	50. 2‐methyl−2‐butenal	51.3‐(1‐ethoxyethoxy)‐butyraldehyde	52. 2‐methoxy‐phenol
53.bicyclo[4.2.0]octa−1,3,5‐triene	54.1,3,5,7‐cyclooctatetraene	55. trimethyl‐pyrazine	56. 3,*N*‐dihydroxy‐butanamide	57. acetamide
58. 3‐(methylthio)−1‐propanol	59. 3‐methyl−2‐butanol	60. benzeneacetic acid, ethyl ester	61. 3‐hydroxy‐butanoic acid, ethyl ester	62. 2‐methyl−3‐oxo‐butanoic acid, methyl ester
63. 2‐pentanol, acetate	64. 5‐methyl−2‐hexanone	65. 4‐s‐butoxy−2‐butanone	66.(2‐ethoxy−1‐methoxyethoxy)‐ethene	67. trimethyl‐oxazole
68. 5‐amino−3‐phenylpyrazole	69. 2,3‐dimethylpyrazine			

As shown in Table 2, in fermented cocoa beans, the main aroma compounds were esters and alcohols. Butyrolactone was more abundant in the fresh beans than that of fermentation samples, the amount of 3‐methyl, 1‐butanol‐acetate was gradually reduced and that of formic acid, 1‐methylpropyl ester was gradually increased. The species of acetate compounds was abundant since it was here that the formation of the characteristic chocolate flavor occurred. According to Adler and others (Adler et al., 2014), the formation of acetate is key step in the development of desirable flavors.

The 2‐pentanol, 3‐methyl‐1‐butanol, 2, 3‐butanediol, and phenylethyl alcohol were the most abundant compounds (Table 2). Only two compounds, 2, 3‐butanediol and benzyl alcohol, gradually increased during the fermentation process, while 2‐pentanol, 2‐heptanol, and 3‐methyl‐1‐butanol decreased. The main flavors of fruity and floral aromas were appeared after fermentation. This fruity aroma (as well as floral aromas) may also be due to the presence of higher alcohols produced during fermentation. These results were similar to those described previously (González, Pérez, & Palomino, 2012). One of the chemical groups that was present in highest abundance in the consummation treatments were acids, representing 56.04% of the total extracted area, followed by alcohols (22.95%) and ketones(9.40%). Other abundant compounds were ester, pyrazines, aldehydes, and terpenoids.

The acidification of cocoa beans by acetic acid during fermentation leads to various biochemical modifications necessary for cocoa flavor development. The content of acetic acid was closely correlated to beans’ pH, suggesting its essential role in cocoa beans’ acidification (Hinneh et al., 2018). In this study, the results showed that the content of acetic acid increased significantly from 4.1% to 33.9% (Table 2). The fruity aroma in cocoa was strongly related to its acidity and increased over the course of fermentation. The result is in agreement with previously reported studies (Gutiérrez, 2017). The content of acetic acid increased was related to the formation of the other aroma compounds such as esters and higher alcohols (Chetschik et al., 2018). The metabolism of the cocoa pulp substrate produces significant amounts of ethanol, acetic acid, and heat, and the description was similar to this study (Adler et al., 2014). The carbohydrate fermentation reactions mainly generate acids, followed by alcohols and ketones. The acidification of cocoa beans by acetic acid during fermentation leads to the formation of various volatile components (alcohols, esters, and acids). These changes include the generation of amino acids from storage proteins by the action of cocoa seed proteases (Apriyanto, 2017). The research showed that the part free amino acids were converted to the cocoa‐ and nutty‐specific aroma components during the heating process. The pyrazine compounds were increased (Voigt, Textoris‐Taube, & Wostemeyer, 2018). It was consistent with the results of this article.

Table 3 showed which compounds in fresh beans disappeared by fermentation and which compounds not founded in fresh beans gradually produced after fermentation. Fermenting leads to the development of specific cocoa aromas via the degradation of proteins, and formation of volatile components, such as pyrazines, which were described as one of the few classes of compounds with desirable flavor properties. The fermentation time of cocoa beans seems to be the key factor controlling the synthesis of aromas, such as methylpyrazine (Hinneh et al., 2018). There were 32 volatile aroma compounds in fresh beans that disappeared after fermentation. Thirty‐seven new compounds were identified in fermented beans. The aroma analysis showed abundant flavor ingredients in fermented beans, in comparison to unfermented samples. Significantly higher concentrations of acids, alcohols, and ketones were found in the beans during fermentation (Tables 2 and 3). The 2, 3‐dimethylpyrazine was recognized as exhibiting a cocoa‐like odor in synergy with 2‐phenyl‐2‐butenal. The fermented beans contained higher amounts of compounds, most likely due to higher levels of precursors synthesized through fermentation. This study showed that samples with longer fermentation time had better cocoa flavor. This result was in agreement with the finding from Hinneh et al. The characteristic cocoa fragrance was found in fermented beans. The well‐fermented cocoa beans gave rise to more compounds and a higher response value than the initial stage of fermentation.

### 3.3 Changes in temperature, color, fat, and protein during the fermentation of different stages

**Figure 1:**
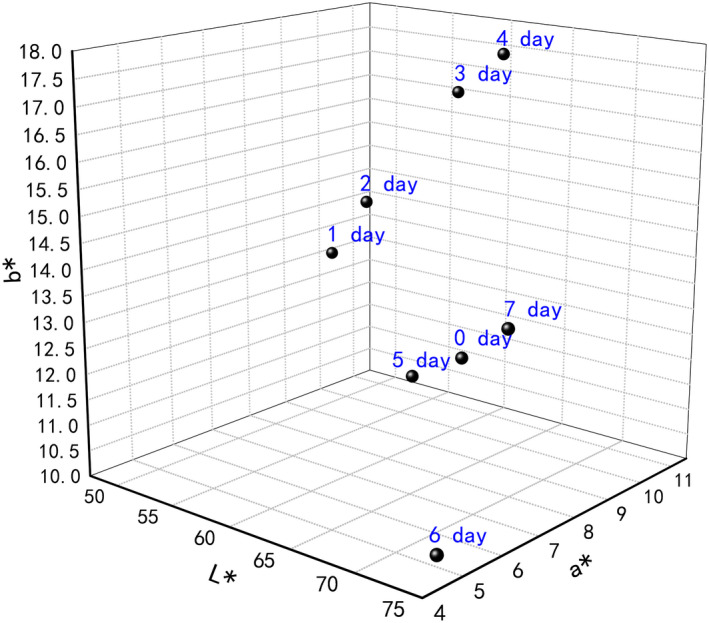
Change (in tristimulus L*, a* and b* values) of the chroma in the cocoa samples (the samples were marked as 0 to 7 days) fermented during different stages. The 3‐dimension graphic represents the difference of the samples

During fermentation, there were also significant color changes that occur in the cocoa beans (Harrington, 2011). To gain a quality change of how the cocoa fermentation affected the color of cocoa beans, measurements of selected colors were conducted in beans at different time points of the fermentation. The 3D stereogram of the tristimulus L*, a*, and b* values of cocoa beans is shown in Figure 1.

The results are expressed in the L*, a*, and b* colorimetric system (Figure 1). The values of L* ranged from 51.38 (1 day) to 73.24 (7 days), 66.79 (0 day). The different L*, which was higher than the other stages, means that the cocoa beans from the fermented 7 days were closer to white on the L*,a*, and b* scale, a* values ranged from 10.62 (4 days) to 4.94 (7 days). All of a* values were positive, which meant the cocoa beans had more of a red color to them. This red color could have been caused by some of the purple beans, which were less fermented than others. The b* values ranged from 17.51 (4 days) to 10.25 (7 days). All of the b* values were positive, which meant they had more of a yellow color instead of blue. The beans that were fermented for 4 days had the highest b* value, so they had the most yellow color of all the fermentation stages. The yellow and brown colors were associated with anthocyanins in the cocoa beans, and the color continued to drop because it showed a decrease in one type of antioxidants as the temperature increased (Harrington, 2011). The colors of the beans in different fermentation stages were different, from deep purple to deep red‐brown due to the level of anthocyanins present. The beans (internally and externally) are dark violet at the beginning of fermentation and turn to dark brown or a dark reddish color by the end (Camu et al., 2008). Therefore, the result from the analysis of procyanidin was considered (Tran et al., 2015). Unfermented beans are originally a gray color and become purple‐brown during fermentation and then become a rich dark brown once the fermentation is complete.

**Figure 2:**
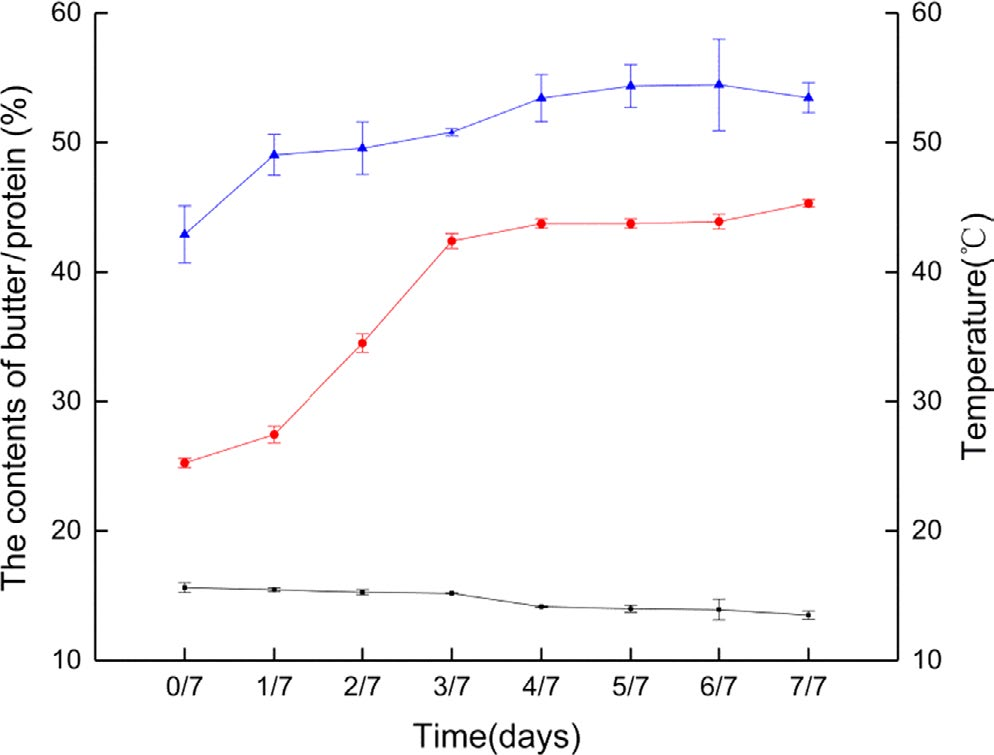
Changes in temperature, protein and fat during the fermentation of different stages: temperature (○), fat (△) and protein (□)

The completion temperature of fermentation was more than 20°C above the initial temperatures due to the internal temperature increase from placing the beans into the wooden box. As the fermentation process continues, the temperature increased (up to 45°C, Figure 2). Cocoa butter was chocolate's main component ingredient; therefore, the change in the content of cocoa butter may directly affect the product's final physical properties, such as melting properties (Campos & Marangoni, 2014). The levels of protein and fat in cocoa beans changed at different times and temperatures. Cocoa proteins underwent extensive hydrolysis during fermentation with consequent increases in peptide abundances, and the amino acids were produced more abundantly as the fermentation progressed (Buyukpamukcu et al., 2001). The cocoa butter contents ranged from 42.90% to 54.45%. The protein contents ranged from 15.63% to 13.51%, and the fermented sample had the highest cocoa butter content. These results were consistent with the above report. The content was comparable to that of cocoa butter (43.44%) as reported (Gu et al., 2013). The protein contents were similar to the protein concentrations in beans from the Dominican Republic (11.8%–15.7%) (Bertazzo, Comai, Brunato, Zancato, & Costa, 2011). It is clear that the migration of acetic acid into the bean combined with the rise of the temperature results in the degradation of storage proteins and carbohydrates into free amino acids.

### 3.4 Effect of cocoa bean fermentation on denaturation

**Figure 3:**
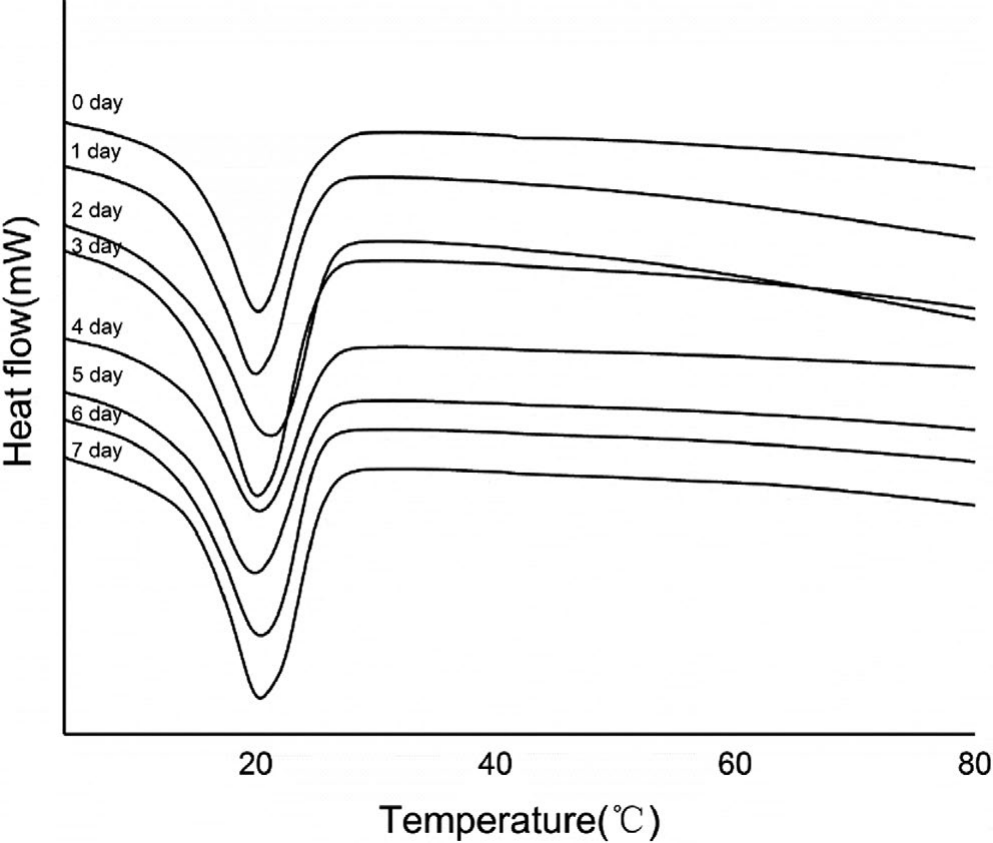
Melting temperatures for several cocoa beans (the samples were marked as 0 to 7 days) as determined by DSC

Thermoanalytical methods, such as DSC, are considered to be convenient and reliable for studying such changes (Drissi, Eddhahak, Caré, & Neji, 2015). The significant exothermic peak can be found in the DSC curve. The analysis spectrogram is shown in Figure 3.

Onset temperatures (*T*_o_) ranged from 5.47 to 7.51°C, denaturation temperatures (*T*_d_) ranged from 12.94 to 14.46°C, peak temperatures (*T*_p_) ranged from 19.87 to 20.97°C, and the value of denaturation enthalpy (Δ*H*) ranged from 30.4 (J/g) to 43.38 (J/g). The value of denaturation enthalpy (Δ*H*) was increased and then decreased with fermentation. The 3‐day fermented sample had the highest Δ*H* (43.38 J/g). The bean products were routinely subjected to various thermal treatments during harvesting, processing and preparation. Thermal treatments may result in the denaturation of protein in cocoa beans (Colombo et al., 2010). The processes by which the internal temperature of the beans were increased and that induce the loss of proteins are presumably the reason for the change of heat flow. Heat treatments have been reported to alter the physical and functional properties of foods. The high thermal sensitivity of some soluble proteins causes other changes in functional properties, including changes in color, flavor, viscosity, texture, or nutritional value. Heating treatments could better product quality including integrity, flavor, and nutrient retention (Llave, Fukuda, Fukuoka, Shibata‐Ishiwatari, & Sakai, 2018).

When the denaturation temperature of 7‐day fermented sample is 14.46°C, the corresponding values of a* and b* were 4.94 and 10.25, respectively. During the fermentation of cocoa beans, the denaturation temperature increased while the color gradually dropped. The content of cocoa butter was increased while the content of protein was reduced as the denaturation temperature increased. As a result, the color of 7‐day fermented beans was deep brown resulting from the combined temperatures and denaturation.

### 3.5 Effects of different fermentation stages on procyanidin extraction yield

**Figure 4:**
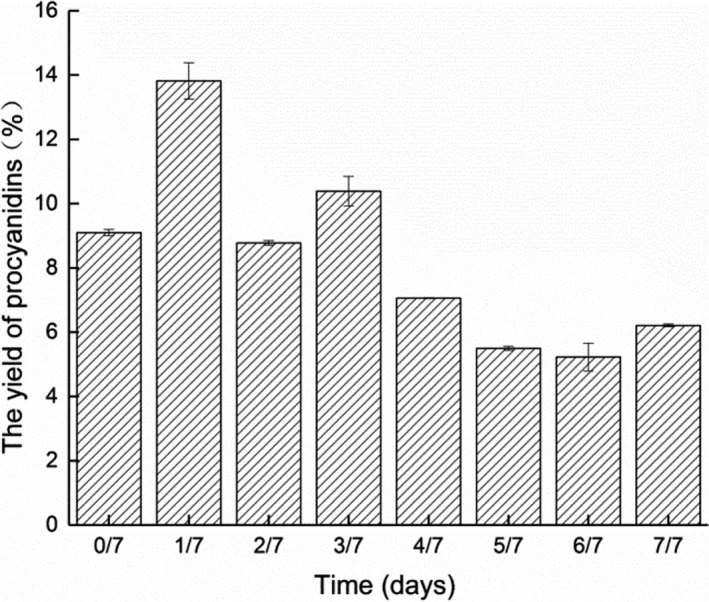
Effects of different fermentation stages on procyanidin extraction yield (%)

It was reported that procyanidin, the main class of polyphenols in cocoa products, imparts not only astringency and bitterness to cocoa but also its exceptional antioxidant activity (Counet, Ouwerx, Rosoux, & Collin, 2004). The change of extraction yield determined on different fermentation days was shown in Figure 4. Standard curve equation: *y* = 7.076 x＋0.0038, *R* ^2^ = 0.9981.

The extraction yield of procyanidin was the highest on Day 1 of the fermentation. As the fermentation process continued, the yields of procyanidin ranged from 13.8% to 6.2%. During fermentation, the procyanidin concentration of beans decreased because of diffusion through water release and through further oxidation and condensation of the polyphenol compounds (Nazaruddin, Seng, Hassan, & Said, 2006). The gradually increased temperature during fermentation may cause a decrease in the procyanidin concentration of the beans. Roasting treatment accompanied by increased temperature significantly reduced the level of polyphenols, including procyanidin (Gültekin‐Özgüven, Berktaş, & Özçelik, 2016). The fermentation also led to a significant reduction of soluble polyphenols, and various volatile compounds (alcohols, esters, and aldehydes) were generated (Hinneh et al., 2018). A previous study showed a link between polyphenols and cocoa flavor intensity (Bariah, 2014). Polyphenols substance contents were significantly lower in under‐fermented and fully fermented beans when compared to raw beans (Tchouatcheu et al., 2019). The content of procyanidin was comparable to those (12.89%) of cocoa bean reported by Cienfuegos et al (Cienfuegos‐Jovellanos et al., 2009). The yields were slightly different from previous studies, which may be because the source and process environment were different.

### 3.6 Effects of different fermentation stages on antioxidant activity

Cocoa polyphenols are antioxidant compounds with beneficial health properties. The antioxidant capacity of foods can be assessed by ABTS or DPPH, the most common being 2, 2′‐azinobis (3‐ethylbenzothiazoline‐6‐sulfonate) (ABTS) and 2, 2′‐diphenyl‐1‐picrylhydrazyl (DPPH) assays (Caporaso, Whitworth, Fowler, & Fisk, 2018). The percentage of scavenged DPPH or ABTS^＋^ was plotted versus the different times of fermentation and the concentration of antioxidant required to obtain 50% inhibition (50% inhibition concentration or IC_50_) was obtained from the graph (Tang & Liu, 2007).

**Figure 5:**
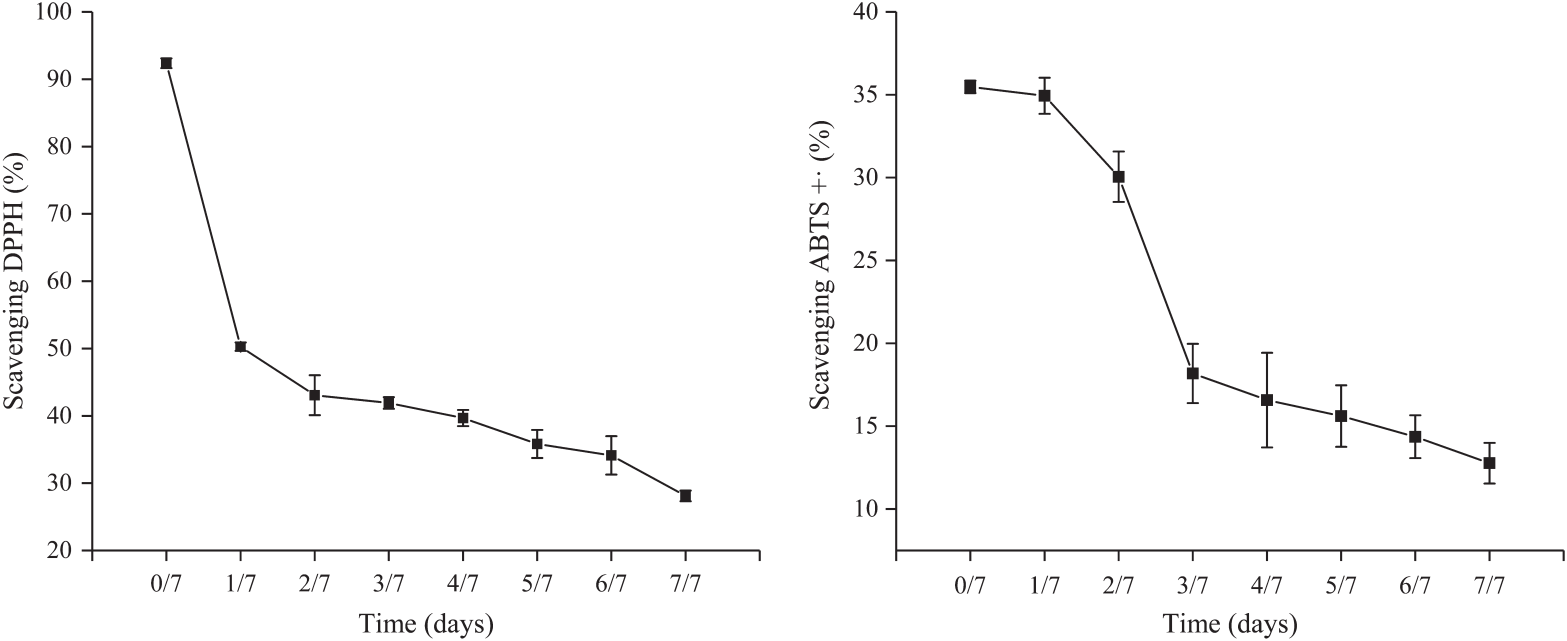
The determination of IC_50_ of cocoa beans, in which the left graph indicated the scavenging DPPH, whereas the right graph exhibited the scavenging ABTS^＋^

The scavenging ability of cocoa bean to hydroxyl radical was shown in Figure 5. Raw bean has the strongest scavenging ability to hydroxyl radical. The scavenging ability of cocoa bean decreased with the fermentation process continued. The change of DPPH scavenge yield determined on different fermentation days was shown in Figure. At the same concentration, the IC_50_ values (μg/ml) of the samples were as follows: 29.680, 60.817, 77.192, 80.020, 130.342, 161.643, 176.442, and 258.835, and the IC_50_ value (μg/ml) of trolox control group was 26.866. During fermentation, the antioxidant capacity of beans gradually reduced. The change of ABTS^＋^ scavenge yield determined on different fermentation days was shown in Figure. The trolox equivalent (μmol/g) of the samples was as follows: 133.554, 131.075, 108.589, 53.935, 46.521, 42.106, 36.347, and 28.992. Antioxidant capacity is strictly correlated to polyphenol content. Raw cocoa had the highest amount of antioxidant activity. During fermentation, the ability of scavenge ABTS^＋^ gradually decreased. The content of total phenolic and procyanidin decreased during fermentation may cause a decrease in the antioxidant capacity of the beans (Gültekin‐Özgüven et al., 2016).

## 4 CONCLUSIONS

The temperature increased gradually during cocoa fermentation, and the protein was degraded gradually to amino acids. The fat content increased slightly, and the colors of the beans were from deep purple to deep red‐brown in fermentation stages while a decrease was accompanied in the procyanidin content and antioxidant activity. Procyanidin was oxidized during fermentation, which reduced the astringency and bitterness of the beans, enhancing the fragrance of the cocoa beans. The fermentation of the beans promoted the formation of the characteristic fragrance of cocoa beans. The fermentation reactions mainly generate acids, followed by alcohols, esters, and ketones. The study showed that primary processing of cocoa beans had a significant effect on the quality formation. Different growing conditions, Genotype, climate, and harvest conditions, as well as processes, such as fermentation and drying all affect the quality of cocoa beans. More work need to be done on describing the quality changes of cocoa beans during fermentation.

## CONFLICT OF INTEREST

The authors have declared no conflicts of interest for this article.

## ETHICAL STATEMENT

This study does not involve any human or animal testing.
